# CMR demonstration of multiple morphological phenotypes in Anderson-Fabry disease

**DOI:** 10.1186/1532-429X-17-S1-Q68

**Published:** 2015-02-03

**Authors:** Djeven P Deva, Kate Hanneman, Qin Li, Paaladinesh Thavendiranathan, Chantal Morel, Robert M Iwanochko, Andrew Crean

**Affiliations:** 1 Medical Imaging, St Michael's Hospital, Toronto, ON, Canada; 2 Cardiology, Toronto General Hospital, Toronto, ON, Canada; 3 Medical Imaging, Toronto General Hospital, Toronto, ON, Canada; 4 Fred A. Litwin Family Centre in Genetic Medicine, University Health Network, Toronto, ON, Canada; 5 Cardiology, Toronto Western Hospital, Toronto, ON, Canada

## Background

We conducted a review of cardiovascular magnetic resonance studies performed in patients with Anderson-Fabry disease (AFD) at our institution aiming to describe the spectrum of imaging findings in this rare disease.

## Methods

All patients with confirmed AFD who had cardiac MRI at our center were included. Short-axis steady state free precession cines and segmented inversion recovery late gadolinium enhancement (LGE) images were acquired using standard parameters. Offline analysis was performed for LV volumes and maximum end-diastolic wall thickness (EDWTmax). Patients were categorized into 4 groups: 1) no wall thickening (EDWT≤12mm); 2) concentric hypertrophy (EDWTmax>12mm with septal to lateral wall thickness ratio <1.3); 3) asymmetric hypertrophy (EDWTmax >12mm with septal to lateral wall thickness ratio ≥1.3); and 4) apical hypertrophy. LGE was quantified using a semi-automated technique with thresholds of 2SD, 4SD and 6SD. Charts were reviewed for clinical information.

## Results

Forty-one patients were included (53.6% male, n=22), median age 45.3 years (range 22.3-68.1). Morphological MR findings are summarized in Table 1. Males were more likely to have increased LV wall thickness and LV mass. The 4-SD threshold for LGE demonstrated the best agreement with manual threshold quantification (*k*=0.62). 6-SD and 2-SD demonstrated lower agreement with manual threshold quantification *k*=0.55 and *k*=0.05 respectively.

**Table 1:** Cardiovascular magnetic resonance findings in Anderson-Fabry disease

	Males (n=22)	Females (n=19)	p-value
Age (years)	47.4 (36.5-55.5)	44.9 (34.9-60.8)	0.896

Extracardiac involvement	21 (95.5%)	15 (78.9%)	0.164

Prior enzyme replacement therapy	17 (77.3%)	11 (57.9%)	0.313

EDWTmax (mm)	14.6 (13.2-17.2)	11.6 (8.9-13.5)	0.013

No wall thickening	4 (18.2%)	12 (63.2%)	0.005

Concentric hypertrophy	14 (63.6%)	5 (26.3%)	0.027

Asymmetric septal hypertrophy	3 (13.6%)	1 (5.3%)	0.610

Apical hypertrophy	1 (4.5%)	1 (5.3%)	1.000

LV ejection fraction (%)	58.3 (54.2-63.0)	60.4 (57.3-65.6)	0.200

LV end diastolic volume index (ml/m2)	92.8 (72.1-111.3)	83.3 (76.2-92.0)	0.151

LV mass index (g/m2)	78.0 (64.8-94.7)	56.7 (51.5-63.0)	0.001

Elevated LV mass index	9 (40.9%)	4 (21.1%)	0.200

Myocardial scar	16 (72.7%)	13 (68.4%)	1.000

Typical midwall lateral wall scar	6 (27.3%)	8 (42.1%)	0.346

Patients with concentric hypertrophy and typical midwall lateral wall scar	5 (22.7%)	4 (21.1%)	1.000

Patients with concentric hypertrophy and typical midwall lateral wall scar Scar as percentage of total myocardium at 4SD calculated from 16 males and 13 females with myocardial scar (%)	4.9 (2.0-10.8)	1.9 (1.2-10.2)	0.381

Age is presented as median (range). All other continuous variables were compared with a Mann-Whitney Rank Sum test and presented as median (interquartile range). Categorical variables were compared with a Fisher test.

Just over two thirds of patients had late gadolinium enhancement (LGE), but only half of these had typical lateral wall mid-myocardial scar. Only a quarter of patients had both concentric wall thickening and typical lateral wall scar. Only 11 of 25 patients with wall thickening had elevated LVMI. There was significantly more myocardial scar (as a percentage of total myocardium) in patients with elevated LVMI (n=10, 10.97%, IQR 7.57-17.43%) than in patients with normal LVMI (n=19, 1.87%, IQR 1.15-4.37%, p<0.001), and significantly more scar in patients with wall thickening (n=21, 7.1%, IQR 2.35-11.71%) than those without wall thickening(n=8, 1.19%, IQR 0.83-2.23%, p=0.003). Patients with elevated LVMI had higher incidence of arrhythmia (atrial fibrillation & ventricular tachycardia[VT]) than those without elevated LVMI (8/13 vs. 3/28, p=0.001). Patients with elevated LVMI had higher incidence of VT than those without elevated LVMI (4/13 vs. 1/28, p=0.028).

## Conclusions

Concentric thickening and lateral wall mid-myocardial scar are the most common manifestations of AFD, but the spectrum includes cases identical to apical and asymmetric septal hypertrophy subtypes of hypertrophic cardiomyopathy.

## Funding

None.

**Figure 1:**
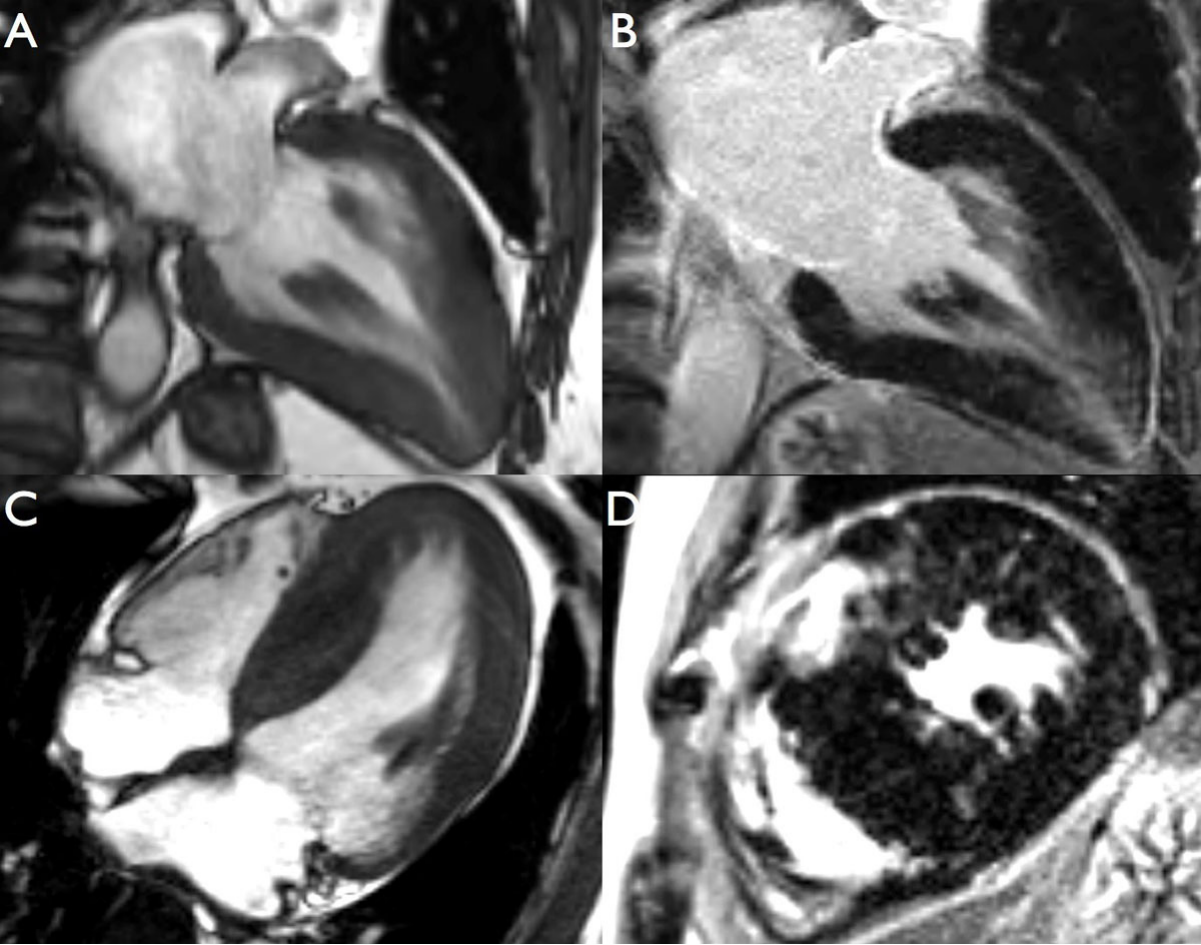
A-B. 63 year old female with Anderson-Fabry Disease on enzyme replacement therapy and a history of ventricular tachycardia. CMR reveals an apical pattern of hypertrophy and apical scar. C-D. 58 year old male with Anderson-Fabry Disease on enzyme replacement therapy. CMR reveals reverse septal curvature subtype of asymmetric septal hypertrophy and predominantly hinge point scar

